# Unveiling novel insights in prostate cancer through single-cell RNA sequencing

**DOI:** 10.3389/fonc.2023.1224913

**Published:** 2023-09-08

**Authors:** Wenyue Yu, Chun Wang, Zhiqun Shang, Jing Tian

**Affiliations:** Tianjin Institute of Urology, Second Hospital of Tianjin Medical University, Tianjin, China

**Keywords:** prostate cancer, ScRNA-seq, tumor microenvironment, tumor heterogeneity, cell interactions

## Abstract

Single-cell RNA sequencing (scRNA-seq) is a cutting-edge technology that provides insights at the individual cell level. In contrast to traditional bulk RNA-seq, which captures gene expression at an average level and may overlook important details, scRNA-seq examines each individual cell as a fundamental unit and is particularly well-suited for identifying rare cell populations. Analogous to a microscope that distinguishes various cell types within a tissue sample, scRNA-seq unravels the heterogeneity and diversity within a single cell species, offering great potential as a leading sequencing method in the future. In the context of prostate cancer (PCa), a disease characterized by significant heterogeneity and multiple stages of progression, scRNA-seq emerges as a powerful tool for uncovering its intricate secrets.

## Introduction

Prostate cancer is currently the most prevalent cancer diagnosed in men within the United States, accounting for 29% of all diagnoses in 2023 and ranking as the second leading cause of cancer-related deaths in men of all ages in 2020 (1). Globally, there are approximately 1.3 million new cases of prostate cancer reported each year (2). It is widely recognized that prostate cancer exhibits significant heterogeneity, both clinically and at the molecular and morphological levels (3). Consequently, comprehensive assessment of this disease often proves challenging when relying solely on molecular analyses of tissue samples as a whole. Thankfully, the advancement of technology has allowed for substantial progress in the field of cancer research, enabling us to explore the genomic landscape and developmental processes of prostate cancer in greater detail.

Among the rapidly evolving technologies, scRNA-seq has emerged as a prominent tool. Leveraging specialized technological expertise and sophisticated bioinformatic analysis, scRNA-seq enables us to investigate gene expression at the individual cell level. By employing mature analytical approaches such as clustering, trajectory inference, cell-type annotation, and dataset integration (4), scRNA-seq provides us with novel dimensions to comprehend prostate cancer in terms of its plasticity, metastatic potential, and tumor microenvironment (TME).

**Figure 1:**
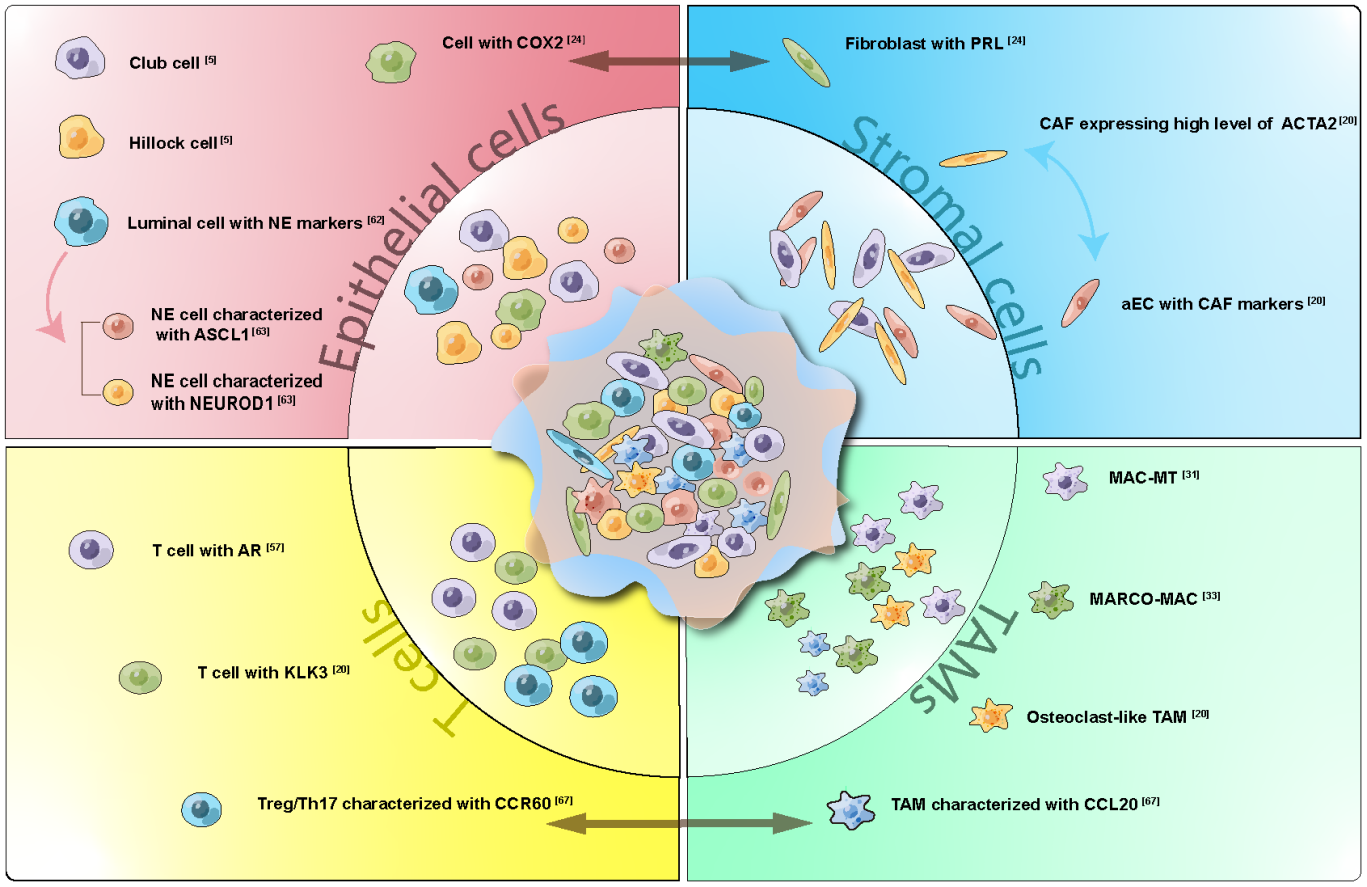
Cell Subtypes, Interactions, and Development Trajectory Revealed by ScRNA-seq in Prostate Cancer. Representative subgroups within Epithelial cells, Stromal cells, T cells, and Tumor Associated Macrophages (TAMs) were delineated. Cellular trajectories were depicted through one-way arrays, while interactions were visualized using two-way arrays. scRNA-seq has proven instrumental in identifying less prevalent cell subtypes and enhancing our understanding of cellular communication and lineage connections within prostate cancer.

This review aims to highlight the latest findings in the field of prostate cancer uncovered through scRNA-seq (**Figure 1**). By examining each stage of prostate cancer progression from a single-cell perspective, and drawing insights from a selection of recently published representative articles, we will explore the impact of this emerging technology on reinterpreting earlier research and shaping the future landscape of prostate cancer study.

## Single-cell RNA sequencing on normal prostate

Traditional fluorescence-activated cell sorting (FACS) has been widely used for cell type identification in tissues. However, it has limitations in detailed cell sorting and discovering new subtypes due to gating strategies and limited antibody availability. In a pioneering study, Henry et al. employed single-cell RNA sequencing to comprehensively profile the transcriptome of the normal adult human prostate. By combining their findings with immunofluorescence and flow cytometry, they achieved the first cellular anatomy of the normal human prostate, establishing a baseline for future prostate disease studies (5).

The anatomical composition of the prostate was originally defined into four main regions: peripheral zone, central zone, preprostatic zone, and anterior fibromuscular stroma by McNeal in 1968 (6). Based on gene expression, cellular location, and surface antigens, three types of epithelial cells have been described in the prostate: basal, luminal, and neuroendocrine (NE) (7–11). Henry et al.’s findings were consistent with previous knowledge, with the addition of two epithelial cell subtypes expressing low levels of both basal and luminal markers (luminal KLK3+ and basal KRT14+). These subtypes were labeled as “other epithelia” (OE), further divided into “OE1” and “OE2”. OE1 was characterized by high expression of SCGB1A1, PIGR, MMP7, CP, and LCN2, while OE2 expressed KRT13, SERPINB1, and CLDN4 (5). By comparing these subtypes with epithelial cells from the mouse lung, they found that OE1 exhibited similarities to lung Scgb1a1+ clara (club) cells and Krt13+ hillock cells, whereas OE2 showed similarities to lung Krt5+ basal and Krt13+ hillock cells (5, 12, 13).

The localization of different cell types was further examined using *in situ* triple immunofluorescence. OE1 cells (SCGB1A1+) were predominantly found in the prostatic urethra and collecting ducts, but were rare in the prostate itself. On the other hand, OE2 cells (KRT13+) were abundant in the prostatic urethra, collecting ducts, and the central zone surrounding the ejaculatory ducts, but were scarce in the peripheral zone (5).

Previous studies have indicated low expression of KRT family genes in the adult prostate, with enrichment observed in fetal prostate and localized prostate tumors (14). Thus, the KRT13+ OE2 cell type was suggested to be associated with tumorigenesis (5). The specific function of OE1 cells is yet to be elucidated.

The study also identified stromal components of the prostate, including smooth muscle cells, fibroblasts, and leukocytes. This application of scRNA-seq on human prostate represents a significant milestone in prostate research, as it demonstrated the technology’s impressive ability to identify previously unrecognized subtypes. Although the study did not explicitly identify neuroendocrine cells, possibly due to their scarcity, subsequent research has addressed these limitations and further explored the biological functions of the newly discovered subgroups.

## Primary prostate tumors

Building upon the discoveries made by Henry et al., Song et al. aimed to investigate the representation of epithelial diversity within primary prostate tumors (15).

Using scRNA-seq, they examined the profiles of four pairs of tumors and their corresponding benign-appearing tissue. The analysis revealed the presence of basal and luminal cells, consistent with epithelial populations. Additionally, they identified a subset of cells exhibiting lower scores for basal epithelial (BE) and luminal epithelial (LE) signatures, but displaying elevated expression of PIGR, MMP7, and CP. These cells closely resembled the previously reported OE1 cells, as indicated by their expression of SCGB1A1, PIGR, MMP7, CP, and LCN2, as documented by Henry et al. (5). Notably, the identified markers expressed by these OE1 cells have been linked to the generation and progression of tumors (16–18). Consequently, it was inferred that these OE1-like cells may be associated with the process of carcinogenesis.

Due to the observed similarities between OE1 cells in the prostate and club cells in the lung (5), the analogous cells discovered by Song et al. were designated as “club cells” in their study. To further investigate the role of club cells in PCa, the researchers categorized both tumor and normal samples’ club cells into six distinct cell states labeled as club 0 to club 5. Among these clusters, club 0 was found to be significantly enriched in the tumor group. This cluster exhibited upregulation of LTF, luminal markers, and a range of downstream genes related to the androgen receptor (AR) pathway. Enrichment analysis also revealed a substantial enrichment in the Hallmark Androgen Response pathway. Another cluster, club 6, which was also enriched in the tumor group, displayed similar characteristics. Collectively, these findings suggested a luminal-like and androgen-responsive state of PCa epithelial cells (15).

Further investigation led to the identification of LE and BE subclusters characterized by the expression of club cell markers (initially proposed for normal prostate cancer by Henry et al., as mentioned above, encompassing SCGB1A1, PIGR, MMP7, CP, and LCN2 (5)). These subclusters were referred to as “club cell states.” Supported by additional scRNA-seq datasets (19, 20), a set of PCa-club cell signatures was established, including PIGR, LTF, and NKX3-1 (15).

It is noteworthy that in their investigation, Song et al. did not observe the presence of OE2-like cells (also referred to as “hillock cells” due to their similarity to hillock cells in the lung) within their PCa samples (5). However, these cells were identified in PCa organoids. This disparity suggests that hillock cells may experience depletion during the progression of prostate cancer. Despite this intriguing observation, further research is required to unravel the underlying mechanisms responsible for this phenomenon and provide a more comprehensive explanation (15).

Song et al.’s study elucidated the heterogeneity of epithelial cells in prostate cancer, specifically investigating the functional characteristics of club and hillock cells within PCa. Their research significantly expanded upon the previous findings of Henry et al.

Additionally, Song et al. conducted a study aiming to elucidate the distinctions attributed to TMPRSS2-ERG fusion, a prevalent oncogenic transcription factor in PCa. Tumor cells exhibiting elevated ERG expression were categorized as ERG+, while cells lacking ERG expression were designated as ERG-. Notably, there was no noteworthy disparity in copy number variations (CNV) between ERG+ and ERG- tumor cells. However, variations were observed in terms of inter-tumor heterogeneity and transcriptomic profiles. Firstly, ERG+ cells exhibited a patient-specific clustering pattern, unlike ERG- cells. Secondly, ERG- cells displayed a greater overlap in gene expression with non-malignant LE cells and occupied a closer proximity to LE on the UMAP map, indicating a higher degree of similarity (15). Moreover, ERG- patients demonstrated increased shared signaling pathways between T-cells and stromal cells, and both CD4 and CD8 cells associated with ERG- tumor cells displayed signs of exhaustion and a cytotoxic phenotype, thereby suggesting a promising potential for immunotherapy in clinical trials (15). In conclusion, these findings suggest that the overexpression of ERG in PCa epithelia contributes to increased intra- and inter-cellular heterogeneity and is associated with unfavorable outcomes.

The study conducted by Chen et al. provides a novel perspective on the heterogeneity of PCa epithelia.

The researchers analyzed epithelial cells obtained from 13 tissue samples of PCa, including 12 primary tumors and 1 lymph node metastasis. Through the utilization of PAM50, a gene expression signature initially discovered in breast cancer, the epithelial cells were classified into three subtypes: luminal A, luminal B, and basal (16). Most of the subclusters exhibited characteristics of both luminal A and luminal B subtypes, with the exception of clusters 10 and 12. Cluster 10 demonstrated a high expression signal for luminal A genes but also expressed gene markers associated with basal/intermediate cells. Therefore, it was designated as “Basal/Intermediate cells” (20). Conversely, cluster 12 displayed a high expression signal for luminal B genes and exhibited an overexpression of genes involved in cell-cycle phases, leading to its designation as “CellCycle” (20). Survival analysis using data from the TCGA database revealed that patients with the CellCycle signature had a worse prognosis, consistent with previous studies (21). On the other hand, patients with Basal/Intermediate signatures exhibited a better outcome, partially aligning with the findings of Song et al. that PCa epithelia display a more luminal-like state (15). Chen et al. also investigated the immune aspect of the findings. They discovered that basal/intermediate cells showed high expression of antigen processing and presentation genes (20), specifically expressing HLA class II and the chemokine gene CCL2, which could enhance the immune response against tumor cells and potentially contribute to better survival. Additionally, basal/intermediate cells involved in perineural invasion (PNI) were found to have significant communication with neural cells through the CXCL and CCL signaling networks (22). This communication was identified as a critical factor in promoting cancer progression and metastasis in PNI-PCa (22). Furthermore, a separate study involving primary tumor tissues from 14 untreated PCa patients reported that the epithelial subcluster with the highest number of cycling cells exhibited the highest epithelial-to-mesenchymal transition (EMT) score, indicating greater tumor ecosystem diversity, which was associated with a poorer prognosis (23).

The heterogeneity observed among epithelial tumor cells can often be attributed to the diverse interactions occurring within the tumor tissue. Utilizing scRNA-seq, Zheng et al. identified a distinct subtype of tumor cells derived from micro-metastases of prostate cancer in mice. This subtype displayed notable differences compared to tumor cells found at primary sites and exhibited a high expression of prolactin receptor (Prlr) (24). Building upon this discovery, subsequent experiments revealed a significant intercellular interaction pathway: tumor cells exhibited elevated levels of COX-2 (prostaglandin endoperoxide synthase 2), which facilitated the synthesis of PGE2. This, in turn, stimulated the secretion of prolactin by upregulating nuclear receptor 4A (NR4A) in stromal cells. The increased concentration of prolactin subsequently induced the expression of PRLR in neighboring tumor cells, thereby promoting early metastasis in prostate cancer (24). The identification of this new tumor cell subtype unveiled the important role played by COX-2 in the progression of prostate cancer. Furthermore, when combined with the findings of Peng et al., which highlighted the various immunosuppressive effects derived from EP4, a high-affinity receptor of PGE2 (25), it can be inferred that COX-2 inhibitors could serve as meaningful additions to clinical therapies.

The heterogeneity observed in PCa extends beyond epithelial cells and also encompasses the stromal compartment.

Fibroblast and endothelial cells (ECs) are recognized as vital components of the TME. The interactions between cancer-associated fibroblasts (CAFs) and tumor cells, as well as tumor-infiltrating immune cells, have been identified as key factors in promoting tumor progression (26). In the study conducted by Chen et al., it was observed that ACTA2, a gene encoding actin proteins, which was reported to be decreased in CAFs of PCa due to changes in stromal composition (27), exhibited abundant expression in CAFs. Through correlation analysis, researchers suggested its association with EMT (20). Transcription factor (TF) analysis revealed a subset of CAFs enriched in genes involved in the extracellular matrix (ECM), leading to their characterization as “activated CAFs”. Genes related to activated CAFs were also expressed in certain subsets of ECs, which were labeled as “aECs.” These particular aECs were found to be enriched in castration-resistant prostate cancer (CRPC) and exhibited the ability to modify the ECM and downregulate immune activation through cell-cell interactions (20).

CAFs that are enriched in invasive prostate cribriform carcinoma (ICC) and intraductal carcinoma (IDC) were found to exhibit elevated expression levels of CTHRC1, ASPN, FAP, and ENG. This upregulation of genes in CAFs was hypothesized to be associated with dysfunctional T cells (28).

The roles of immune cells in tumor progression have garnered increasing attention in recent years. The use of scRNA-seq to characterize the diverse population of infiltrating immune cells in PCa provides valuable insights for a better understanding of this phenomenon.

Tumor-associated macrophages (TAMs) play significant roles in regulating tumorigenesis (29). While macrophages have traditionally been viewed as anti-tumor cells involved in immune defense, numerous studies have highlighted the dual nature of TAMs. Specifically, TAMs have been shown to stimulate angiogenesis, enhance tumor cell migration and invasion, and suppress antitumor immunity, thereby contributing to the progression of tumors towards malignancy (29, 30).

In a scRNA-seq study involving 10 pairs of human cancer and adjacent normal tissue, a distinct subset of TAMs characterized by high expression of metallothionein family genes, particularly zinc transporter genes SLC30A1 (ZNT-1) and SLC39A8 (ZIP-8), was identified. These TAMs were labeled as “MAC-MT” (31). Since zinc is a crucial component of prostatic fluid, the MAC-MT subset was considered a prostate-specific macrophage subtype that plays a role in maintaining zinc metabolism homeostasis (32). Importantly, the MAC-MT subset exhibited increased expression of IFNγ-, THF-, as well as CXCL9 and CXCL10, indicating its association with anti-cancer immune response and potentially contributing to better patient outcomes (31).

In addition to TAMs associated with zinc metabolism, TAMs linked to lipid metabolism have also been discovered. Through clustering analysis of macrophages from three treatment-naïve patients, two distinct macrophage subtypes, Mac1 and Mac2, were identified. Comparison of tumor and adjacent nontumor tissues revealed that both Mac1 and Mac2 subtypes increased in tumor tissues and were associated with pathways related to lipid metabolism, with Mac2 showing a more pronounced association (33). Further investigation focused on Mac2 revealed that TAMs characterized by the high expression of MARCO exhibited a significant correlation with lipid metabolism and could serve as a prognostic signature (33).

TAMs can also serve as a predictive factor for metastasis. Chen et al. identified a specific subset of macrophages characterized by a high expression level of osteoclast (OC)-related pathways, including mineral absorption and lysosome functions (20). This finding is particularly significant considering that prostate cancer commonly metastasizes to bone, where osteoclasts play crucial roles, and inhibiting osteoclast activity has been shown to delay bone metastasis in prostate cancer (34). The discovery of this subset of TAMs suggests that the potential for metastasis may already be present in the early stages of the tumor (20).

Tumor-associated macrophages (TAMs) characterized by the M2 markers CD163 and MSR1 in ICC and ICD were found to exhibit upregulated expression of C1QB, TREM2, and APOE. Importantly, the presence of these TAMs was associated with worse progression survival outcomes (28).

T cells play a crucial role in TME and serve as indicators of immune activity. In PCa, the TME is generally considered to be immunosuppressive, as evidenced by the proportion of regulatory T cells (Tregs) and the expression of cytotoxic genes in CD4+ and CD8+ T cells (31, 33). However, subsets of T cells within PCa tissue samples have been found to express high levels of KLK3, the gene encoding prostate-specific antigen (PSA) (20). These KLK3-high T-cell clusters exhibit specific modules associated with extracellular vesicles (EVs) and exosomes, suggesting that the abundance of KLK3 might be attributed to EVs derived from tumor cells. This hypothesis was confirmed through subsequent experiments, which demonstrated that T cells expressing KLK3 contribute to the establishment of a pre-metastatic niche for tumor cells (20). Furthermore, single-cell proteomics analysis involving 58 prostate cancer patients with different International Society of Urological Pathology (ISUP) grades revealed that T cells were enriched in the high-grade sub-cohort and exhibited a highly proliferative phenotype (35).

## Oncogenesis

The process of tumor formation, known as oncogenesis, has been a subject of extensive discussion. One theory proposes that tumors originate from a specific group of stem cells (36), while another suggests that a majority of cells can contribute to tumorigenesis. To gain insights into the underlying mechanisms of oncogenesis in PCa, researchers have employed scRNA-seq technology.

Karthaus et al. conducted an experiment to investigate the stemness potential of mouse prostate epithelia during androgen deprivation and restoration, focusing on the regeneration of the prostate. The epithelial cells were classified into seminal vesicle subsets, basal subsets, and three luminal subsets referred to as luminal 1, 2, and 3 cells (L1, L2, L3). Among these luminal subpopulations, L1 cells constituted the largest proportion. L1 cells exhibited high expression of canonical androgen receptor target genes (such as Pbsn, Nkx3.1) and genes associated with mature secretory cells (CD26/Dpp4+, DC59a, and CD133/Prom 1). L2 cells were characterized by the expression of Scal/Ly6a, Tacstd2/Trop2, and Psca, which have been reported to be involved in stem cell-like activity. L3 cells expressed the transcription factor Foxil, a master regulator of subunits of the vacuolar ATPase proton pump, suggesting a potential association between the L3 subset and epididymal fluid acidification (19). Furthermore, regarding their spatial distribution, L1 cells were primarily located in the distal prostate ducts, L2 cells were predominantly found in the proximal prostate region, and L3 cells were interspersed in both proximal and distal locations (19).

In order to identify potential stem cells during the regeneration process following androgen deprivation, scRNA-seq data from mouse prostate across a castration/regeneration (C/R) cycle was collected and analyzed. Interestingly, despite L1 cells remaining the majority population, they exhibited striking similarities to L2 cells after castration. However, during the regeneration phase, the expression pattern of L1 cells reverted back to their baseline state, indicating the loss of androgen receptor-regulated transcription. Notably, all three subsets (L1, L2, and L3) displayed an increase in proliferation markers during the regeneration process. The stem-like potential of both L1 and L2 cells was further confirmed through organoid culture, where organoids derived from these subsets gave rise to Krt5+ basal cells, indicating their bi-lineage potential (19).

Furthermore, during the process of regeneration, stromal cells exhibited increased expression of growth factor ligands, such as Nrg2, Igf1, Fgf10, and Rspo3. Simultaneously, the expression of corresponding receptors, including Fgfr2 and Lgr4, was enhanced in L1 cells. This reciprocal interaction between stromal cells and luminal cells indicated the influence of the microenvironment on luminal proliferation and highlighted the importance of cell-cell circuits in this process (19).

These findings suggest that the potential for self-renewal is present in the majority of luminal cells, including the well-differentiated secretory cells of the L1 subset, during the castration/regeneration cycle. This challenges the conventional belief that self-renewal ability is limited to rare stem cells and indicates that almost all luminal cells have the capacity for redifferentiation (19).

In a separate research study focused on identifying a potential population with luminal stem/progenitor properties during prostate homeostasis and regeneration, researchers proposed an alternative theory.

Cells derived from freshly dissociated whole prostate tissue of healthy adult male mice were classified into 11 distinct cell clusters, including three clusters of luminal cells denoted as Luminal-A/B/C (37). Among these subclusters, Luminal-A/B was characterized by the expression of genes associated with iron homeostasis and fluid secretion, suggesting their functional maturation and differentiation (37). Meanwhile, the Luminal-C cluster exhibited high expression levels of Tacst2, Psca, and Ck4. The protein product of Tacstd2 has been identified as a stem cell marker (38), while Psca is known as a tumor antigen associated with prostate cancer (39). Pathway enrichment analysis revealed a significant enrichment in tissue development and epithelial cell differentiation within the Luminal-C clusters (37). Moreover, cell trajectory analysis demonstrated a trajectory from Luminal-C to Luminal-A and Luminal-B, indicating the potential progenitor role of Luminal-C cells (37). This conclusion was supported by co-immunofluorescence experiments. Notably, a subset of Luminal-C cells located at the distal prostate glandular invagination tips, referred to as Dist-Luminal-C, showed high expression levels of Tastd2 and played a crucial role in prostate generation and potential tumorigenesis (37).

It should be noted that the study conducted by Guo et al. exhibited notable similarities to the research conducted by Karthaus et al. Both studies focused on the mouse prostate, conducted castration/regeneration assays, and identified a cluster of cells (Luminal-C in Guo’s study and L2 in Karthaus’ study) with high expression levels of Tastd2 and Psca. However, despite these similarities, different conclusions were drawn between the two studies. The underlying mechanism behind these divergent findings remains to be further explored.

In addition to androgen, vitamin D is a well-known factor associated with prostate cancer. The active form of vitamin D, 1,25(OH)2D3, interacts with its receptor, the vitamin D receptor (VDR), to exert pleiotropic effects in mammals, including proliferation, differentiation, and apoptosis (40–42). Studies have reported that vitamin D deficiency increases the risk of prostate cancer (43), while men with higher plasma 25(OH)D levels in the highest quartile have been found to have less than half the risk of lethal prostate cancer compared to those with lower levels in the lowest quartile (44). The mechanisms underlying the preventive effect of vitamin D on prostate cancer are currently being investigated (45).

McCray et al. conducted a study using organoids derived from the benign region of the human prostate. ScRNA-seq was performed on organoids cultured with sufficient 1,25D (active form of vitamin D) and control organoids at day 8 and day 14, respectively. At day 8, the organoids cultured with sufficient 1,25D exhibited an increase in dividing cells compared to the control group. However, at day 14, the 1,25D-treated organoids showed an increase in polarized and basal cells, as well as a decrease in progenitor and intermediate cells, in comparison to the control group (46). Additionally, the organoids cultured with 1,25D at day 14 showed a higher percentage of cells expressing high levels of Integrin. These findings suggested that 1,25D could promote both growth and differentiation in the prostate. Transcriptome analysis revealed that the promotion of growth and differentiation by 1,25D was primarily achieved by inhibiting the Wnt pathway through upregulation of the dickoff family number 3 (DKK3) gene (46).

In another study, mice with Pten-deficient PIN were utilized to investigate the effects of the vitamin D analog Gemini-72 on prostatic precancerous lesions. ScRNA-seq analysis revealed that the down-regulated genes in response to Gemini-72 treatment were primarily involved in the encoding of collagen proteins, extracellular matrix (ECM) proteins, and ECM remodeling enzymes, suggesting that the main impact of Gemini-72 was on ECM remodeling (47). Epithelial cells were also influenced by Gemini-72 treatment. Among the subclusters of epithelial cells, the luminal-C subcluster characterized by Tacstd2, Krt4, and Ly6a exhibited a significant reduction in proportion after one week of treatment with Gemini-72. However, the proportion of luminal-A/B cells remained similar between the treatment and control conditions. Further subcluster analysis of the Luminal-C cells identified a particularly sensitive cell group characterized by high transcript levels of SASP components, which accumulate in cells undergoing cellular senescence (47). Additionally, persistent subsets of luminal-C cells exhibited upregulated NF-γB pathway activity, which is associated with antiapoptotic processes. These findings suggest that vitamin D analogs such as Gemini-72 may have potential benefits in the prevention and treatment of prostate cancer, as they eliminate precancerous cells through apoptotic cell death (47).

## Castration resistance

Prostate cancer is commonly treated with castration, either through surgical or medical means, as the first-line treatment (48, 49). Initially, castration therapy demonstrates an efficiency rate of 80-90% (50, 51). However, a significant challenge arises as nearly all patients treated with castration therapy eventually develop castration-resistant prostate cancer (52). Therefore, it is crucial to explore the mechanisms underlying castration resistance in order to better understand the progression of prostate cancer.

The utilization of scRNA-seq has proven effective in constructing a longitudinal landscape of prostate cancer progression. Bolis et al. conducted a study using patient-derived xenografts (PDX), providing valuable insights into the trajectory of tumor cell progression during castration. The tumor model exhibiting rapid castration resistance displayed significant differences in single-cell transcriptional profiles before and after castration. A consistent shift was observed across all subsets, characterized by a suppression of canonical AR signaling and an upregulation of pro-proliferation genes associated with MYC (53). This shift across all cell subpopulations supports Karthaus et al.’s conclusion that plasticity potential exists in all prostate cells (19). However, it is worth noting that the PDX cells used in this study were derived from patients with castration-resistant prostate cancer (CRPC). The selection of stem-cell-like subsets may have already occurred due to prior therapy targeting the androgen receptor. Consequently, the conclusion may lack sufficient convincing power.

Another study associated with castration induced tumor cells is the study conducted using Pten^fl/fl^ mice (54). Researchers identified a specific group of cells characterized by the expression of Krt4, Tacstd2, and Ppp1r1b, which were named as intermediate cells (54). These intermediate cells demonstrated a propensity for survival and diversification under castration conditions. Genes that were upregulated in castrated intermediate cells, such as ATP1B1, BST2, CP, IGFBP3, and PTTG1, have been shown to be associated with resistance to ADT in human tumors (54).

In addition, castration was found to induce perturbations in the tumor microenvironment (TME). Following castration, there was an increase in the abundance of tumor-associated macrophages with M2-like characteristics. This was accompanied by a reduction in M1-like features, including TNFα signaling and inflammatory signatures (53).

After profiling the trajectory of PCa at the single-cell level, the study took a further step. By analyzing high-throughput transcriptional datasets from 13 studies, the researchers identified EZH2, a member of the polycomb-repressive complex-2, as the most regulated gene during tumor progression (53). To unravel how EZH2 inhibition restores transcriptional output in CRPC progression, they utilized the EZH2 protein inhibitor GSK126 in LNCaP cells cultured in charcoal-stripped serum (CSS) to investigate the role of EZH2 in PCa progression under castration conditions. Remarkably, the introduction of co-targeting AR and EZH2 resulted in the formation of a completely new subgroup marked by heightened AR signaling and diminished expression of E2F-related cell cycle genes. This underscores that the inhibition of EZH2 within an androgen-depleted context resulted in a near-complete rewiring of transcriptional patterns. Additionally, in xenograft tumors on mice, inhibition of EZH2 delayed the regrowth of LNCaP xenografts. ScRNA-seq profiles demonstrated that GSK126 treatment led to an augmentation in the least progressed subcluster, indicating a reversal of progression along the trajectory. Furthermore, a decrease in M2-like macrophages was observed in GSK126-pretreated tumors. These findings highlight the crucial role of EZH2 in castration-resistant PCa progression and introduce a novel potential for utilizing EZH2 inhibition in clinical treatment for CRPC progression (53).

To investigate the significance of the androgen receptor (AR) in castration resistance, He et al. conducted a study involving the collection of biopsies from three metastatic sites (bone, lymph node, and liver) before and after enzalutamide therapy. ScRNA-seq was utilized to exclude the influence of cells from the metastatic sites. By comparing the transcription profiles before and after castration therapy, the researchers observed an overall upregulation of AR and its isoforms, which was expected (55).

The study aimed to explore the relationship between specific AR isoforms and castration resistance. However, no individual AR isoform exhibited any relevance, even when examining paired biopsies from the same patient before and after therapy (55). The increased expression of AR isoforms was more likely a consequence of the overall increase in total AR expression, rather than playing an independent role in castration resistance (55).

Nevertheless, there were other differences observed due to castration aside from AR isoforms. Cells subjected to castration displayed a higher enrichment of gene sets associated with epithelial-mesenchymal transition (EMT) and transforming growth factor (TGF)-β signaling. Furthermore, castration therapy also influenced the immune microenvironment (55).

It has long been recognized that the majority of prostate tumors are not responsive to immune checkpoint inhibitors (56). Further investigation of T cells provided additional evidence in this regard. Several markers associated with exhaustion and dysfunction, such as PDCD1, HAVCR2, TOX, TIGIT, ICOS, FASLG, and LAG3, were observed, aligning with expectations (55). Additionally, clonotype groups of T cells detected in metastases exhibited genes related to co-inhibitory receptors and CXCR4, which may contribute to the unresponsiveness to immune checkpoint inhibitors (ICI/ICB) (55).

Another study focused on the association between checkpoint blockade and the efficacy of T cells. Guan et al. conducted research involving eight patients with metastatic castration-resistant prostate cancer (mCRPC), among whom three patients responded to pembrolizumab (defined as a prostate-specific antigen (PSA) decline of >25% upon immune checkpoint blockade), while five did not respond (57). ScRNA-seq was conducted on metastatic tumor lesions obtained from patients. The CD8+ T cells from these patients formed two clusters referred to as CD8 k1 and CD8 k2. Remarkably, these two clusters closely overlapped with CD8 T cell clusters divided by responders (CD8 R) and non-responders (CD8 NR). Analysis of differentially expressed genes between CD8 R and CD8 NR revealed a deactivation of AR in CD8 R (57). Inspired by the findings from scRNA-seq, a series of experiments were conducted, unveiling that AR could bind to open chromatin regions associated with Ifng and Gzmb, thereby causing immune suppression. These bindings could be suppressed by enzalutamide. The combination of androgen deprivation therapy (ADT) and enzalutamide enhanced the effect of PD-1/PD-L1 blockade in mice (57).

In the context of longitudinally exploring cancer progression, scRNA-seq provides a novel perspective for investigating the lineage and evolution of tumor cells. By employing cell trajectory analysis, it becomes possible to uncover hidden subtypes that may play a dominant role in tumor progression. This approach allows for a deeper understanding of the cellular dynamics and heterogeneity within the tumor, contributing to a more comprehensive characterization of cancer progression.

Taavitsainen et al. conducted a study utilizing PCa cell lines to investigate the mechanism underlying drug castration resistance. They performed both scRNA-seq and single-cell transposase-accessible chromatin sequencing (scATAC-seq) on LNCaP parental cell lines and LNCaP-derived enzalutamide (ENZ) cell lines. The scRNA-seq profiles were largely consistent with the scATAC-seq data, characterized by high expression of genes associated with AR and MYC, indicating that the transcriptional changes induced by drug castration were primarily driven by chromatin reprogramming (58). The cells were classified into different clusters, and certain clusters, referred to as “persist clusters,” showed no significant change in their percentage before and after castration. The persist clusters exhibited high proliferative activity, and some of them demonstrated relatively high expression of genes associated with stemness (58). Trajectory analysis revealed that these cells possessed high developmental potential and could give rise to other clusters. They were characterized by a gene signature named “Persist”. Genes associated with regenerative mouse prostate luminal 2 cells, as reported by Karthaus et al., were extracted (19). Among these, 78 genes with human homologs were designated as the “PROSGenesis signature”. When assessing clusters using the PROSGenesis signature, cluster 10 in the parental cell line before treatment displayed the highest score. Subsequent trajectory analysis demonstrated that this cluster served as a precursor to castration-induced clusters (58). Scores of xenografts from AR+/NE−, AR−/NE+, or AR−/NE− CRPC and NEPC tumors resistant to ENZ revealed that the PROSGenesis signature exhibited notably high scores in AR+ tumors. Among primary treatment-naive patients, a high PROSGenesis score was linked to an extended response to ADT, potentially attributed to the heightened influence of AR activity within these tumors. Moreover, clinical data revealed that the PROSGenesis signature was associated with longer progression-free survival (PFS), and cells with a high PROSGenesis signature score predominantly belonged to the basal/intermediate subtype (58). This finding further supports the results from Chen et al., who reported that basal/intermediate cells were indicative of longer recurrence-free survival (20).

Cheng et al. conducted a study using clinical samples to define pre-existing castration-resistant subpopulations in primary prostate cancer, specifically in castration-resistant prostate cancer classified as adenocarcinoma (CRPC-adeno) and small cell neuroendocrine carcinoma (SCNC or CRPC-NE or NEPC). They performed scRNA-seq on specimens from three cases of primary adenocarcinoma and three castration-resistant PCa (including two CRPC-adeno and one NEPC). Based on the trajectory analysis generated by Monocle, non-basal cells followed two main directions: AR-dependent CRPC-adeno trajectory and an AR-independent NEPC trajectory. Surprisingly, in addition to cells derived from CRPC/NEPC samples, a small number of primary PCa cells were also classified into CRPC/NEPC clusters (59). These cells were identified as either NE or CRPC-like cells based on their gene expression profiles. The trajectory analysis of NEPC progression revealed that NE cells from primary PCa were distributed along the trajectory, indicating their self-renewal capability and ability to evolve under the pressure of castration. On the other hand, CRPC-like cells were primarily located at the terminal state of the CRPC trajectory, indicating their advanced progression (59). These newly identified CRPC-like cells were found to exist in multiple databases, and the gene signature derived from them was shown to be meaningful in predicting clinical outcomes (59).

As mentioned earlier, NEPC represents a distinct stage in castration-resistant prostate cancer, characterized by the loss of AR expression and acquisition of neuroendocrine features (16, 60–62). The study conducted by Brady et al. delved into the development and composition of NEPC. Using gene-edited mice, the initial part of the research aimed to confirm that Pten^f/f^; RB1^f/f^; MYCN+ (PRN) mice had a higher propensity to develop NEPC compared to Pten^f/f^; MYCN+ (PN) mice and Pten^f/f^; RB1^f/f^ (PR) mice. This suggested that the presence of MYCN amplification and Rb1 deletion could expedite the progression to castration resistance and NEPC tumors (62). Subsequently, scRNA-seq was performed on both PRN mice and age-matched PR mice. As anticipated, cells derived from PRN mice exhibited a higher proportion of neuroendocrine (NE) cells, with 75% originating from PRN tumors and 25% from PR tumors. Furthermore, these NEPC cells displayed an increased adult stem cell (ASC) signature score (62).

Remarkably, the trajectory analysis conducted in the study unveiled the existence of a distinctive subtype of luminal epithelial cells. Despite being classified as luminal cells, this particular cluster exhibited low expression levels of neuroendocrine markers and was positioned adjacent to the neuroendocrine (NE) cells along the cell trajectories. These luminal cells emerged more frequently and at an earlier stage in pseudotime in PRN tumors compared to PR tumors. Additionally, they shared an expression module with NE cells, suggesting their role as precursors to NE cells (62).

Furthermore, the study identified two distinct subclusters within the neuroendocrine (NE) cells, distinguished by the expression of Ascl1 and Pou2f3, respectively (62). This finding is somewhat consistent with the research conducted by Cejas et al., which focused on treatment-emergent neuroendocrine prostate cancer (NEPC) using patient-derived xenograft (PDX) models. Cejas et al. classified NEPC into two subtypes, but based on the expression of ASCL1 and NEUROD1 rather than ASCL1 and POU2F3 (63). Moreover, another study involving seven patients with prostate cancer identified two NEPC gene expression signatures: NE1 characterized by ASCL1, and NE2 marked by GHGA, GHGB, and ENO2 (64). These discrepancies in subtype classification may be attributed to differences in the sample species (gene-edited mice vs. PDX models) and potential batch effects. Ultimately, further research is needed to precisely define the subtypes of NEPC.

Additionally, the study highlighted two axes of NEPC evolution from androgen-dependent prostate cancer (ADPC). One axis was driven by genetic events, which involved gains in 12q, losses in 15q, amplification of MYC, and losses in RB1. The other axis was influenced by transcription factors. Specifically, NKX2-2 was identified as a key transcription factor at the early stage of NEPC development, while POU3F2 and SOX2 played crucial roles at the late stage (64). These findings provide insights into the molecular mechanisms underlying the transition from ADPC to NEPC, with both genetic alterations and transcriptional regulation contributing to the evolution of NEPC.

## Migration

Bone metastases, a severe complication of cancer, have the potential to be life-threatening. In the context of prostate tumors, bone metastases are often regarded as an advanced stage and are observed in approximately 90% of men who experience treatment-resistant disease (65–67). Typically associated with castration resistance and immunosuppression (68), PCa with bone metastasis is commonly considered incurable. The underlying mechanisms behind this phenomenon have been extensively examined and debated.

Intercellular communication within the tumor microenvironment (TME) plays a crucial role in regulating immunity and has significant implications in bone metastasis. In a study conducted by Owen et al., scRNA-seq techniques were employed to investigate the dormancy and proliferation of PCa cells residing in bone metastases. Differential gene expression analysis revealed an enrichment of interferon-regulated genes (IRGs) in dormant cells, with type I interferons (IFN-α/β) being the most prominent (67). Notably, the clusters of dormant cells exhibited significant upregulation of genes associated with positive regulation of immune cells, including lymphocyte activation, as well as antigen processing and presentation. These findings suggest that immune-activating mechanisms in the bone microenvironment contribute to maintaining the dormant state of tumor cells. Conversely, the loss of intrinsic type I interferon signaling was found to promote the proliferation of metastatic tumor cells. Follow-up experiments provided further support for this hypothesis. It was also demonstrated that the suppression of tumor-intrinsic type I interferon signaling is induced by the bone marrow environment, and its reversion enhances the effectiveness of immunotherapy (67). These findings indicate a potential novel therapeutic approach targeting bone metastases.

Further investigation into the relationship between the immune microenvironment and bone migration was carried out. Kfoury et al. conducted a study that elucidated how immunocytes collaborate to establish an immunosuppressive TME. The study utilized scRNA-seq data from patients with spinal metastasis, including samples obtained from the solid tumor, involved vertebral area, and distant vertebral regions, as well as benign controls. This comprehensive analysis provided insights into the immune compartment at both the tumor and liquid bone marrow levels. Differences in cell composition and gene expression profiles were observed between the benign and malignant samples, with the most significant disparities found in myeloid and T cells (69).

Regarding myeloid cells, the malignant fractions exhibited notable enrichment in tumor inflammatory monocytes (TIMs) and TAMs. TIMs demonstrated high expression of genes associated with activation and proliferation, while TAMs displayed characteristics consistent with the M2 phenotype (69). It has been reported that both TIMs and TAMs contribute to tumor progression and inflammation suppression by secreting factors such as epiregulin, EGF, NFKBIA, and TNFAIP3. The presence of these cells is often associated with a poor outcome (70, 71).

In terms of T cells, the tumor fractions demonstrated a significant decrease in the proportions of T cells compared to those found in healthy individuals. Among the cytotoxic T lymphocytes (CTLs), two distinct groups were identified. CTL-1 exhibited gene expression patterns associated with effector T cells, such as KLRG1, GZMK, and other cytotoxic mediators. On the other hand, CTL-2 displayed a transcriptional profile characteristic of effector/memory-like T cells, including genes like IL7R and KLRB1 (69). CTL-2 displayed an overall dysfunctional and exhausted phenotype, while increased activity signatures were observed in regulatory T cells (Tregs) at the metastatic site. These findings collectively indicate an immunosuppressive environment within TME.

Subsequent analysis suggested a correlation between the proportion of TAMs and the exhaustion of CTL-2. This finding implies a communication between the myeloid and lymphoid compartments within the tumor microenvironment. To further validate this observation, the researchers investigated the ligands and cognate receptors involved in the interaction between TIMs/TAMs and T cells. The results revealed that CCL20, expressed by TIMs/TAMs, could recruit Tregs and T_H_17 cells through its cognate receptor CCR6. This recruitment mechanism contributes to an overall immunosuppressive environment in bone metastases (69).

## Discussion

The concept of implementing RNA sequencing at the single-cell level was first demonstrated using neuronal cells in 1992 (72). Through a 15-year evolution, the high-throughput sequencing approach known as 10x Genomics was introduced in 2016, marking a pivotal advancement. This development laid the essential groundwork for the subsequent widespread adoption of single-cell RNA sequencing. The first application of scRNA-seq to prostate research was published in December 2018 (5), which is within a span of less than five years. As with any nascent technique, there are challenges to be faced. A primary hurdle involves sample acquisition and preparation. Given scRNA-seq’s stringent demand for fresh tumor specimens, it has brought difficulties to postoperative pathological examination or disease recurrence observation. This constraint is partially alleviated through nuclear sequencing, viable for frozen or fixed samples. Another challenge pertains to noise. The modest sample size and shallow sequencing depth inherent to scRNA-seq yield extensive sparse and noisy data. Effective processing strategies, encompassing QC (quality control), normalization, imputation, feature selection, and dimensionality reduction, offer partial mitigation (73).

ScRNA-seq has significantly advanced prostate cancer research and clinical assessment by identifying subtypes and gene sets with prognostic implications. Notably, MAC-MT signifies a favorable prognosis (31), while T cells expressing KLK3 serve as metastasis predictors (20), and the PROSGenesis signature correlates with extended PFS (58). Moreover, this technology has unveiled new potential candidates for clinical intervention, such as COX-2 inhibitors (24, 25), Gemini-72 (47), and GSK126 (53). As a promising platform, scRNA-seq offers a higher-resolution landscape, empowering researchers to delve into rare subtypes, unravel tumor cell lineages, and investigate cellular interactions.

Beyond scRNA-seq, the rapid evolution of single-cell multi-omics, encompassing scATAC-seq (single-cell Assay for Transposase-Accessible Chromatin sequencing), single-cell proteomics, single-cell spatial transcriptomics, and single-cell metabolomics, is evident. The dawn of the single-cell omics era for human cancer is imminent, promising a comprehensive grasp of prostate cancer and laying the groundwork for personalized medicine.

The utilization of scRNA-seq, either alone or in combination with other experimental approaches, has significantly contributed to reinforcing and reinterpreting our understanding of prostate cancer within a relatively short timeframe. This rapidly advancing technology has enabled us to explore the heterogeneity of both tumor cells and the TME from a novel perspective. The application of scRNA-seq in prostate cancer research provides us with a precise anatomical understanding of different cell types and reveals the lineage relationships within subpopulations of the same cell type. Furthermore, it allows for the characterization of intercellular communication between different cell types. By subjecting the transcriptome of prostate cancer cells to the scrutiny of scRNA-seq, we can visualize the transcriptomic landscape at the level of individual cells, while at the same time understand tumor as a dynamic and communicative ensemble.

## Author contributions

WY wrote the first draft of the manuscript. ZS, JT, and CW did revision and editing of the manuscript. All authors contributed to the article and approved the submitted version.

## References

[1] Siegel RL Miller KD Wagle NS Jemal A. Cancer statistics, 2023. CA Cancer J Clin (2023) 73:17–48. doi: 10.3322/caac.21763 36633525

[2] Sandhu S Moore CM Chiong E Beltran H Bristow RG Williams SG. Prostate cancer. Lancet (2021) 398:1075–90. doi: 10.1016/S0140-6736(21)00950-8 34370973

[3] Haffner MC Zwart W Roudier MP True LD Nelson WG Epstein JI. Genomic and phenotypic heterogeneity in prostate cancer. Nat Rev Urol (2021) 18:79–92. doi: 10.1038/s41585-020-00400-w 33328650 PMC7969494

[4] Zhang Z Cui F Lin C Zhao L Wang C Zou Q. Critical downstream analysis steps for single-cell RNA sequencing data. Brief Bioinform (2021) 22(5):bbab105. doi: 10.1093/bib/bbab105 33822873

[5] Henry GH Malewska A Joseph DB Malladi VS Lee J Torrealba J. A cellular anatomy of the normal adult human prostate and prostatic urethra. Cell Rep (2018) 25:3530–42. doi: 10.1016/j.celrep.2018.11.086 PMC6411034 30566875

[6] McNeal JE. The zonal anatomy of the prostate. Prostate (1981) 2:35–49. doi: 10.1002/pros.2990020105 7279811

[7] Shen MM Abate-Shen C. Molecular genetics of prostate cancer: new prospects for old challenges. Genes Dev (2010) 24:1967–2000. doi: 10.1101/gad.1965810 20844012 PMC2939361

[8] Foster CS Dodson A Karavana V Smith PH Ke Y. Prostatic stem cells. J Pathol (2002) 197:551–65. doi: 10.1002/path.1194 12115870

[9] van Leenders GJ Schalken JA. Epithelial cell differentiation in the human prostate epithelium: implications for the pathogenesis and therapy of prostate cancer. Crit Rev Oncol Hematol (2003) 46 Suppl:S3–10. doi: 10.1016/S1040-8428(03)00059-3 12850522

[10] Hudson DL. Epithelial stem cells in human prostate growth and disease. Prostate Cancer Prostatic Dis (2004) 7:188–94. doi: 10.1038/sj.pcan.4500745 15289813

[11] Shappell SB Thomas GV Roberts RL Herbert R Ittmann MM Rubin MA. Prostate pathology of genetically engineered mice: definitions and classification. The consensus report from the Bar Harbor meeting of the Mouse Models of Human Cancer Consortium Prostate Pathology Committee. Cancer Res (2004) 64:2270–305. doi: 10.1158/0008-5472.CAN-03-0946 15026373

[12] Hong KU Reynolds SD Giangreco A Hurley CM Stripp BR. Clara cell secretory protein-expressing cells of the airway neuroepithelial body microenvironment include a label-retaining subset and are critical for epithelial renewal after progenitor cell depletion. Am J Respir Cell Mol Biol (2001) 24:671–81. doi: 10.1165/ajrcmb.24.6.4498 11415931

[13] Montoro DT Haber AL Biton M Vinarsky V Lin B Birket SE. A revised airway epithelial hierarchy includes CFTR-expressing ionocytes. Nature (2018) 560:319–24. doi: 10.1038/s41586-018-0393-7 PMC6295155 30069044

[14] Liu S Cadaneanu RM Zhang B Huo L Lai K Li X. Keratin 13 is enriched in prostate tubule-initiating cells and may identify primary prostate tumors that metastasize to the bone. PLoS One (2016) 11:e0163232. doi: 10.1371/journal.pone.0163232 27711225 PMC5053503

[15] Song HA-O Weinstein HNW Allegakoen P Wadsworth MH 2nd Xie J Yang H. Single-cell analysis of human primary prostate cancer reveals the heterogeneity of tumor-associated epithelial cell states. Nat Commun (2022) 13(1):141. doi: 10.1038/s41467-021-27322-4 35013146 PMC8748675

[16] Wang JJ Mao JH. The etiology of congenital nephrotic syndrome: current status and challenges. World J Pediatr (2016) 12:149–58. doi: 10.1007/s12519-016-0009-y 26961288

[17] Zhang Q Liu S Parajuli KR Zhang W Zhang K Mo Z. Interleukin-17 promotes prostate cancer via MMP7-induced epithelial-to-mesenchymal transition. Oncogene (2017) 36:687–99. doi: 10.1038/onc.2016.240 PMC5213194 27375020

[18] Fotiou K Vaiopoulos G Lilakos K Giannopoulos A Mandalenaki K Marinos G. Serum ceruloplasmin as a marker in prostate cancer. Minerva Urol Nefrol (2007) 59:407–11. 17947957

[19] Karthaus WA-O Hofree M Choi D Linton EL Turkekul M Bejnood A. Regenerative potential of prostate luminal cells revealed by single-cell analysis. Science (2020) 368(6490):497–505. doi: 10.1126/science.aay0267 32355025 PMC7313621

[20] Chen S Zhu G Yang Y Wang F Xiao Y-T Zhang N. Single-cell analysis reveals transcriptomic remodellings in distinct cell types that contribute to human prostate cancer progression. Nat Cell Biol (2021) 23(1):87–98. doi: 10.1038/s41556-020-00613-6 33420488

[21] Cuzick J Swanson GP Fisher G Brothman AR Berney DM Reid JE. Prognostic value of an RNA expression signature derived from cell cycle proliferation genes in patients with prostate cancer: a retrospective study. Lancet Oncol (2011) 12:245–55. doi: 10.1016/S1470-2045(10)70295-3 PMC3091030 21310658

[22] Zhang B Wang S Fu Z Gao Q Yang L Lei Z. Single-cell RNA sequencing reveals intratumoral heterogeneity and potential mechanisms of Malignant progression in prostate cancer with perineural invasion. Front Genet (2022) 13:1073232. doi: 10.3389/fgene.2022.1073232 36712886 PMC9875799

[23] Ge G Han Y Zhang J Li X Liu X Gong Y. Single-cell RNA-seq reveals a developmental hierarchy super-imposed over subclonal evolution in the cellular ecosystem of prostate cancer. Adv Sci (Weinh) (2022) 9:e2105530. doi: 10.1002/advs.202105530 35322584 PMC9131431

[24] Zheng Y Comaills V Burr R Boulay G Miyamoto DT Wittner BS. COX-2 mediates tumor-stromal prolactin signaling to initiate tumorigenesis. Proc Natl Acad Sci U.S.A. (2019) 116(12):5223–32. doi: 10.1073/pnas.1819303116 PMC6431196 30819896

[25] Peng S Hu P Xiao Y-T Lu W Guo D Hu S. Single-cell analysis reveals EP4 as a target for restoring T-cell infiltration and sensitizing prostate cancer to immunotherapy. Cli Cancer Res (2022) 28(3):552–67. doi: 10.1158/1078-0432.CCR-21-0299 34740924

[26] Mao X Xu J Wang W Liang C Hua J Liu J. Crosstalk between cancer-associated fibroblasts and immune cells in the tumor microenvironment: new findings and future perspectives. Mol Cancer (2021) 20:131. doi: 10.1186/s12943-021-01428-1 34635121 PMC8504100

[27] Ayala G Tuxhorn JA Wheeler TM Frolov A Scardino PT Ohor M. Reactive stroma as a predictor of biochemical-free recurrence in prostate cancer. Clin Cancer Res (2003) 9:4792–801. 14581350

[28] Wong HY Sheng Q Hesterberg AB Croessmann S Rios BL Giri K. Single cell analysis of cribriform prostate cancer reveals cell intrinsic and tumor microenvironmental pathways of aggressive disease. Nat Commun (2022) 13:6036. doi: 10.1038/s41467-022-33780-1 36229464 PMC9562361

[29] Quail DF Joyce JA. Microenvironmental regulation of tumor progression and metastasis. Nat Med (2013) 19:1423–37. doi: 10.1038/nm.3394 PMC3954707 24202395

[30] Qian BZ Pollard JW. Macrophage diversity enhances tumor progression and metastasis. Cell (2010) 141:39–51. doi: 10.1016/j.cell.2010.03.014 20371344 PMC4994190

[31] Tuong ZK Loudon KW Berry B Richoz N Jones J Tan X. Resolving the immune landscape of human prostate at a single-cell level in health and cancer. Cell Rep (2021) 37(12):110132. doi: 10.1016/j.celrep.2021.110132 34936871 PMC8721283

[32] Costello LC Franklin RB. A comprehensive review of the role of zinc in normal prostate function and metabolism; and its implications in prostate cancer. Arch Biochem Biophys (2016) 611:100–12. doi: 10.1016/j.abb.2016.04.014 PMC5083243 27132038

[33] Masetti M Carriero R Portale F Marelli G Morina N Pandini M. Lipid-loaded tumor-associated macrophages sustain tumor growth and invasiveness in prostate cancer. J Exp Med (2021) 219(2):e20210564. doi: 10.1084/jem.20210564 34919143 PMC8932635

[34] Hayes AR Brungs D Pavlakis N. Osteoclast inhibitors to prevent bone metastases in men with high-risk, non-metastatic prostate cancer: A systematic review and meta-analysis. PLoS One (2018) 13:e0191455. doi: 10.1371/journal.pone.0191455 29370211 PMC5784941

[35] De Vargas Roditi L Jacobs A Rueschoff JH Bankhead P Chevrier S Jackson HW. Single-cell proteomics defines the cellular heterogeneity of localized prostate cancer. Cell Rep Med (2022) 3:100604. doi: 10.1016/j.xcrm.2022.100604 35492239 PMC9044103

[36] Clevers H Watt FM. Defining adult stem cells by function, not by phenotype. Annu Rev Biochem (2018) 87:1015–27. doi: 10.1146/annurev-biochem-062917-012341 29494240

[37] Guo WA-O Li L He J Liu Z Han M Li F. Single-cell transcriptomics identifies a distinct luminal progenitor cell type in distal prostate invagination tips. Nat Genet (2020) 52(9):908–18. doi: 10.1038/s41588-020-0642-1 PMC8383310 32807988

[38] Goldstein AS Huang J Guo C Garraway IP Witte ON. Identification of a cell of origin for human prostate cancer. Science (2010) 329:568–71. doi: 10.1126/science.1189992 PMC2917982 20671189

[39] Reiter RE Gu Z Watabe T Thomas G Szigeti K Davis E. Prostate stem cell antigen: a cell surface marker overexpressed in prostate cancer. Proc Natl Acad Sci U.S.A. (1998) 95:1735–40. doi: 10.1073/pnas.95.4.1735 PMC19171 9465086

[40] Holick MF. Vitamin D deficiency. N Engl J Med (2007) 357:266–81. doi: 10.1056/NEJMra070553 17634462

[41] Feldman D Krishnan AV Swami S Giovannucci E Feldman BJ. The role of vitamin D in reducing cancer risk and progression. Nat Rev Cancer (2014) 14:342–57. doi: 10.1038/nrc3691 24705652

[42] Bikle D Christakos S. New aspects of vitamin D metabolism and action - addressing the skin as source and target. Nat Rev Endocrinol (2020) 16:234–52. doi: 10.1038/s41574-019-0312-5 32029884

[43] Murphy AB Nyame Y Martin IK Catalona WJ Hollowell CMP Nadler RB. Vitamin D deficiency predicts prostate biopsy outcomes. Clin Cancer Res (2014) 20:2289–99. doi: 10.1158/1078-0432.CCR-13-3085 PMC4104275 24789033

[44] Shui IM Mucci LA Kraft P Tamimi RM Lindstrom S Penney KL. Vitamin D-related genetic variation, plasma vitamin D, and risk of lethal prostate cancer: a prospective nested case-control study. J Natl Cancer Inst (2012) 104:690–9. doi: 10.1093/jnci/djs189 PMC3341310 22499501

[45] Fleet JC Kovalenko PL Li Y Smolinski J Spees C Yu J-G. Vitamin D signaling suppresses early prostate carcinogenesis in tgAPT(121) mice. Cancer Prev Res (Phila) (2019) 12:343–56. doi: 10.1158/1940-6207.CAPR-18-0401 PMC7194567 31028080

[46] McCray T Pacheco JV Loitz CC Garcia J Baumann B Schlicht MJ. Vitamin D sufficiency enhances differentiation of patient-derived prostate epithelial organoids. iScience (2021) 24(1):101974. doi: 10.1016/j.isci.2020.101974 33458620 PMC7797919

[47] Abu El Maaty MA-O Grelet E Keime C Rerra A-I Gantzer J Emprou C. Single-cell analyses unravel cell type-specific responses to a vitamin D analog in prostatic precancerous lesions. Sci Adv (2021) 7(31). doi: 10.1126/sciadv.abg5982 PMC8324049 34330705

[48] Schmidt KT Huitema ADR Chau CH Figg WD. Resistance to second-generation androgen receptor antagonists in prostate cancer. Nat Rev Urol (2021) 18:209–26. doi: 10.1038/s41585-021-00438-4 33742189

[49] Hou Z Huang S Li Z. Androgens in prostate cancer: A tale that never ends. Cancer Lett (2021) 516:1–12. doi: 10.1016/j.canlet.2021.04.010 34052327

[50] Trewartha D Carter K. Advances in prostate cancer treatment. Nat Rev Drug Discovery (2013) 12:823–4. doi: 10.1038/nrd4068 24172327

[51] Wang Y Chen J Wu Z Ding W Gao S Gao Y. Mechanisms of enzalutamide resistance in castration-resistant prostate cancer and therapeutic strategies to overcome it. Br J Pharmacol (2021) 178:239–61. doi: 10.1111/bph.15300 33150960

[52] Davies A Conteduca V Zoubeidi A Beltran H. Biological evolution of castration-resistant prostate cancer. Eur Urol Focus (2019) 5:147–54. doi: 10.1016/j.euf.2019.01.016 30772358

[53] Bolis MA-O Bossi D Vallerga A Ceserani V Cavalli M Impellizzieri D. Dynamic prostate cancer transcriptome analysis delineates the trajectory to disease progression. Nat Commun (2021) 12(1):7033. doi: 10.1038/s41467-021-26840-5 34857732 PMC8640014

[54] Germanos AA Arora S Zheng Y Goddard ET Coleman IM Ku AT. Defining cellular population dynamics at single-cell resolution during prostate cancer progression. Elife (2022) 11.:e79076 doi: 10.7554/eLife.79076.sa2 36511483 PMC9747158

[55] He MX Cuoco MS Crowdis J Bosma-Moody A Zhang Z Bi K. Transcriptional mediators of treatment resistance in lethal prostate cancer. Nat Med (2021) 27(3):426–33. doi: 10.1038/s41591-021-01244-6 PMC7960507 33664492

[56] Teo MY Rathkopf DE Kantoff P. Treatment of advanced prostate cancer. Annu Rev Med (2019) 70:479–99. doi: 10.1146/annurev-med-051517-011947 PMC6441973 30691365

[57] Guan X Polesso F Wang C Sehrawat A Hawkins RM Murray SE. Androgen receptor activity in T cells limits checkpoint blockade efficacy. Nature (2022) 606(7915):791–6. doi: 10.1038/s41586-022-04522-6 PMC10294141 35322234

[58] Taavitsainen SA-O Engedal N Cao S Handle F Erickson A Prekovic S. Single-cell ATAC and RNA sequencing reveal pre-existing and persistent cells associated with prostate cancer relapse. Nat Commun (2021) 12(1):5307. doi: 10.1038/s41467-021-25624-1 34489465 PMC8421417

[59] Cheng Q Butler W Zhou Y Zhang H Tang L Perkinson K. Pre-existing castration-resistant prostate cancer-like cells in primary prostate cancer promote resistance to hormonal therapy. Eur Urol (2022) 81(5):446–55. doi: 10.1016/j.eururo.2021.12.039 PMC9018600 35058087

[60] Aparicio A Tzelepi V Araujo JC Guo CC Liang S Troncoso P. Neuroendocrine prostate cancer xenografts with large-cell and small-cell features derived from a single patient's tumor: morphological, immunohistochemical, and gene expression profiles. Prostate (2011) 71:846–56. doi: 10.1002/pros.21301 PMC3883511 21456067

[61] Beltran H Prandi D Mosquera JM Benelli M Puca L Cyrta J. Divergent clonal evolution of castration-resistant neuroendocrine prostate cancer. Nat Med (2016) 22:298–305. doi: 10.1038/nm.4045 26855148 PMC4777652

[62] Brady NA-O Bagadion AM Singh R Conteduca V Emmenis Van L Arceci E. Temporal evolution of cellular heterogeneity during the progression to advanced AR-negative prostate cancer. Nat Commun (2021) 12(1):3372. doi: 10.1038/s41467-021-23780-y 34099734 PMC8185096

[63] Cejas PA-O Xie Y Font-Tello A Lim K Syamala S Qiu X. Subtype heterogeneity and epigenetic convergence in neuroendocrine prostate cancer. Nat Commun (2021) 12(1):5775. doi: 10.1038/s41467-021-26042-z 34599169 PMC8486778

[64] Wang Z Wang T Hong D Dong B Wang Y Huang H. Single-cell transcriptional regulation and genetic evolution of neuroendocrine prostate cancer. iScience (2022) 25:104576. doi: 10.1016/j.isci.2022.104576 35789834 PMC9250006

[65] Nørgaard M Jensen AØ Jacobsen JB Cetin K Fryzek JP Sørensen HT. Skeletal related events, bone metastasis and survival of prostate cancer: a population based cohort study in Denmark (1999 to 2007). J Urol (2010) 184:162–7. doi: 10.1016/j.juro.2010.03.034 20483155

[66] Perrault L Fradet V Lauzon V LeLorier J Mitchell D Habib M. Burden of illness of bone metastases in prostate cancer patients in Québec, Canada: A population-based analysis. Can Urol Assoc J (2015) 9:307–14. doi: 10.5489/cuaj.2707 PMC4662390 26664661

[67] Owen KL Gearing LJ Zanker DJ Brockwell NK Khoo WH Roden DL. Prostate cancer cell-intrinsic interferon signaling regulates dormancy and metastatic outgrowth in bone. EMBO Rep (2020) 21(6):e50162. doi: 10.15252/embr.202050162 32314873 PMC7271653

[68] Xiang L Gilkes DM. The contribution of the immune system in bone metastasis pathogenesis. Int J Mol Sci (2019) 20(4):999. doi: 10.3390/ijms20040999 30823602 PMC6412551

[69] Kfoury Y Baryawno N Severe N Mei S Gustafsson K Hirz T. Human prostate cancer bone metastases have an actionable immunosuppressive microenvironment. Cancer Cell (2021) 39:1464–1478.e1468. doi: 10.1016/j.ccell.2021.09.005 34719426 PMC8578470

[70] Huynh J Etemadi N Hollande F Ernst M Buchert M. The JAK/STAT3 axis: A comprehensive drug target for solid Malignancies. Semin Cancer Biol (2017) 45:13–22. doi: 10.1016/j.semcancer.2017.06.001 28647610

[71] Lawrence T. The nuclear factor NF-kappaB pathway in inflammation. Cold Spring Harb Perspect Biol (2009) 1:a001651. doi: 10.1101/cshperspect.a001651 20457564 PMC2882124

[72] Eberwine J Yeh H Miyashiro K Cao Y Nair S Finnell R. Analysis of gene expression in single live neurons. Proc Natl Acad Sci U.S.A. (1992) 89:3010–4. doi: 10.1073/pnas.89.7.3010 PMC48793 1557406

[73] Zhang Z Cui F Wang C Zhao L Zou Q. Goals and approaches for each processing step for single-cell RNA sequencing data. Brief Bioinform (2021) 22. doi: 10.1093/bib/bbaa314 33316046

